# Tortuosity-powered microfluidic device for assessment of thrombosis and antithrombotic therapy in whole blood

**DOI:** 10.1038/s41598-020-62768-4

**Published:** 2020-04-01

**Authors:** David J. Luna, Navaneeth K. R. Pandian, Tanmay Mathur, Justin Bui, Pranav Gadangi, Vadim V. Kostousov, Shiu-Ki Rocky Hui, Jun Teruya, Abhishek Jain

**Affiliations:** 1 Department of Biomedical Engineering, Texas A&M College of Engineering, College Station, TX USA; 2 Division of Transfusion Medicine & Coagulation, Department of Pathology & Immunology, Texas Children’s Hospital & Baylor College of Medicine, Houston, TX USA; 3 Department of Paediatrics, Texas Children’s Hospital & Baylor College of Medicine, Houston, TX USA; 4 Department of Medicine, Texas Children’s Hospital & Baylor College of Medicine, Houston, TX USA; 5 Department of Medical Physiology, College of Medicine, Texas A&M Health Science Center, Bryan, TX USA

**Keywords:** Thrombosis, Biomedical engineering

## Abstract

Accurate assessment of blood thrombosis and antithrombotic therapy is essential for the management of patients in a variety of clinical conditions, including surgery and on extracorporeal life support. However, current monitoring devices do not measure the effects of hemodynamic forces that contribute significantly to coagulation, platelet function and fibrin formation. This limits the extent to which current assays can predict clotting status in patients. Here, we demonstrate that a biomimetic microfluidic device consisting stenosed and tortuous arteriolar vessels would analyze blood clotting under flow, while requiring a small blood volume. When the device is connected to an inline pressure sensor a clotting time analysis is applied, allowing for the accurate measurement of coagulation, platelets and fibrin content. Furthermore, this device detects a prolonged clotting time in clinical blood samples drawn from pediatric patients on extracorporeal membrane oxygenation receiving anticoagulant therapy. Thus, this tortuosity activated microfluidic device could lead to a more quantitative and rapid assessment of clotting disorders and their treatment.

## Introduction

Several clinical scenarios (ex. surgery, trauma, life support devices etc.) require anticoagulation or platelet therapy and consequently, manage their serious consequences of bleeding or thrombosis^1–3^. Coagulation and platelet function tests, such as, activated clotting time (ACT), activated partial thromboplastin time (aPTT), thromboelastography and platelet aggregometry, are often imprecise, resulting in false positives and false negatives. This aspect limits their ability to predict thrombotic status in clinical settings^4–6^. An underlying, yet critical contributor to the inaccuracies with these tests is that they fail to incorporate the mechanical and biochemical cues that activate clotting *in vivo*. It is well-known that whole blood thrombosis is highly dependent on hemodynamic forces (flow and shear stress) and cellular interactions. For instance, flow acceleration and deceleration, resulting in fluid shear gradients, have been shown to initiate platelet aggregation during arterial thrombosis *in vivo*, and clotting in extracorporeal life support devices usually occurs at sites of sudden flow disturbances, stagnation points and stenosed sections of tubing^7,8^. Recently, a microfluidic assay that mimics a parallel network of stenosed arteriole vessels and exposes whole human blood to pathophysiological shear rates and gradients was demonstrated to more reliably predict thrombosis when compared to standard laboratory tests^9^. However, this device takes more than 1 mL of blood and in some cases, requires more than 20 minutes to complete analysis. Therefore, despite being biomimetic, it may not be well suited for rapid analysis where minimal amount of blood use is available, such as, pediatric applications. Interestingly, tortuous blood vessels have been shown to induce fluid dynamical disturbances and shear gradients that make them hotspots for forming thrombi *in vivo*^[110^. Also, some prior studies have shown increased thrombosis due to tortuosity *in vitro*^11^. Here, we harnessed these biological architectural principles and created a device with the integration of shear-gradients caused by tortuosity to stenosis-like expansion contractions in the microfluidic format. We demonstrate that this newly designed tortuosity-activated assay achieves clotting time within few minutes and consumes approximately 500 µL blood, which is significantly lower than previous microfluidic assays that measure haemostasis or thrombosis. Furthermore, we show the potential of this tortuosity-activated assay for use in thrombin inhibitor dose monitoring, evaluating platelet count and antifibrinolytics. Finally, with this device, we assess blood samples of paediatric patients in critical care who were on extracorporeal membrane oxygenation receiving anticoagulant therapy. Thus, we provide proof-of-feasibility of a versatile tool for potential clinical applications where thrombosis or altered haemostasis in patients is involved.

## Results

### Design criteria of a tortuous microchannel

**Figure 1:**
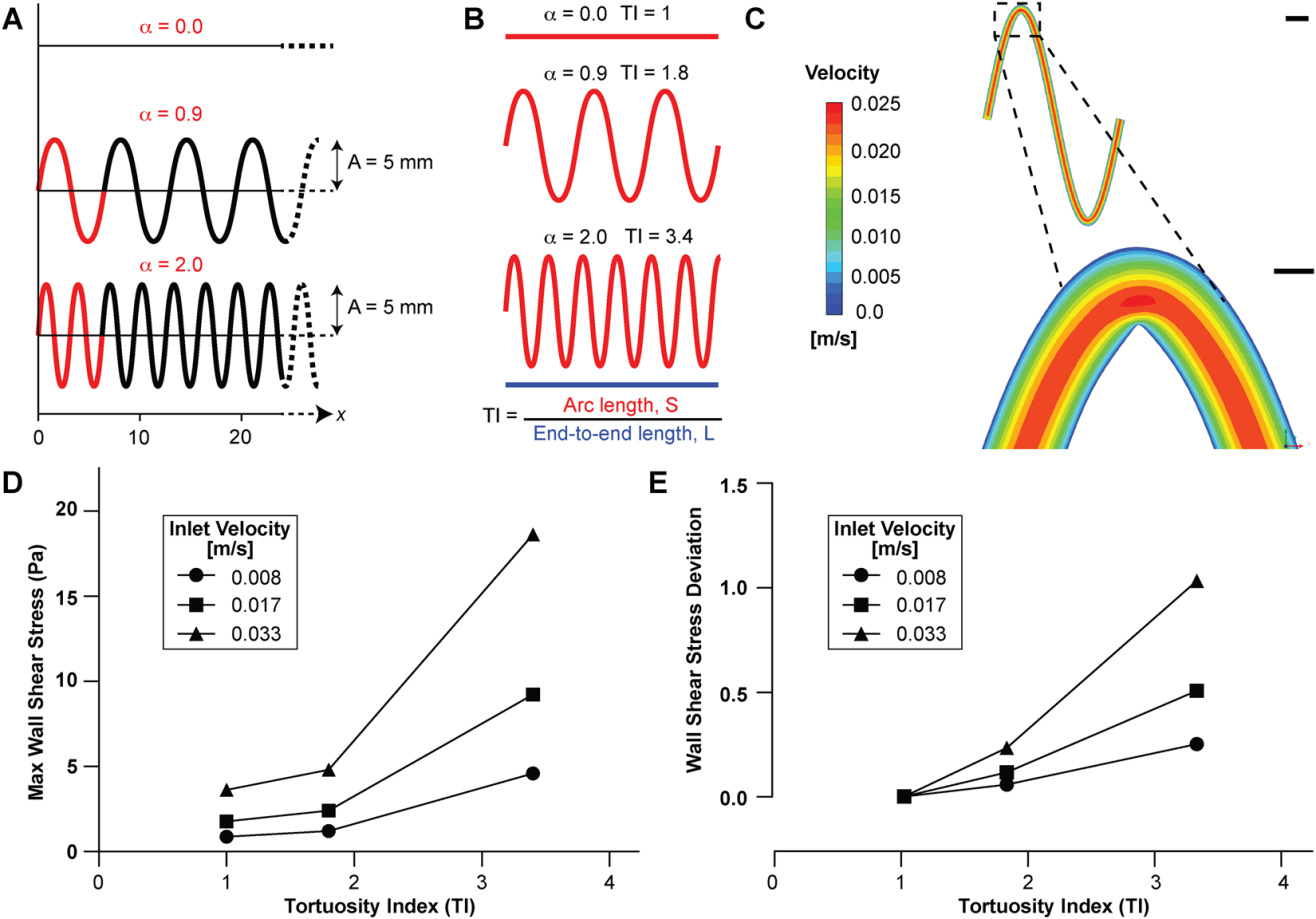
Design and computational analysis of tortuous microchannels. (**A**) The tortuous microchannels were designed in Solidworks (v2019; www.solidworks.com) as sinusoidal curves of varying frequency (α, red), fixed amplitude (A, 5 mm), and fixed total length along x-axis (56 mm). (**B**) Illustration of computed tortuosity index (TI). TI was calculated as the ratio of the arc length (S, red) to end-to-end distance (L, blue, fixed at 56 mm). **(C)** Representative heatmaps showing velocity profile in microchannel of TI = 3.4 at an inlet velocity of 0.017 m/s (scale bar: top, 500 µm; bottom, 100 µm). Graph showing (**D**) maximum wall shear stress (Pa) and **(E)** wall shear stress deviation (fluid shear gradients) as a function of increasing inlet velocities for TI = 1 (circle), 1.8 (square) and 3.4 (triangle). CFD analysis was performed using Ansys (v19.2; www.ansys.com).

We initiated our project by investigating the criteria required in the design of tortuous microchannels. Using the equation of a sinusoidal curve,

1 $$y=A\,\sin (\alpha x)$$

where *A* is the amplitude (mm), α is the frequency (rad/mm), and *x* is the end-to-end length (mm), we generated three microchannel designs in SolidWorks of varying frequency (0, 0.9, and 2.0). The amplitude and end-to-end length were fixed at 5 mm and 56 mm, respectively (Fig. 1A). In this microchannel, we also set the primary dimensions - width, height and length - to be 200 µm, 75 µm and 56 mm respectively, so that it mimics the size a typical arteriole of an equivalent diameter ~100 μm. Also, this configuration is easy to fabricate, image, perfuse with blood, and fits on a standard glass slide. We then applied a commonly used tortuosity index (TI) for blood vessels to these microchannels which is defined as the ratio of vessel arc length over the line distance between the two ends^12^ (Fig. 1B). We calculated the vessel arc length as

2 $$\frac{ds}{dx}=\sqrt{1+{\left(\frac{dy}{dx}\right)}^{2}}=\sqrt{1+{A}^{2}{\alpha }^{2}{(\cos ax)}^{2}}$$

3 $$S={\int }_{0}^{L}ds={\int }_{0}^{L}(\sqrt{1+{A}^{2}{\alpha }^{2}{(\cos ax)}^{2}})dx$$

where *ds* is the infinitesimal arc length, *S* is the arc length, *A* (amplitude) was fixed at 5 mm, and *α* is the frequency. Using equations (2) and (3), TI was then computed as:

4 $$\begin{array}{rcl}{\boldsymbol{TI}} & = & \frac{Arc\,Length}{End\,to\,End\,Length}=\frac{S}{L}\\  & = & \frac{1}{L}{\int }_{0}^{L}(\sqrt{1+{A}^{2}{\alpha }^{2}{(\cos ax)}^{2}})dx\end{array}$$

where *L* (end-to-end distance) was fixed at 56 mm. We found that upon varying frequency, we could design microchannels with a TI ranging from unity (straight channel) to a maximum of 3.4 (30° arc angle), beyond which the channels cannot be further bent. However, this range in TI is typically observed in blood vessels *in vivo*^13^.

### Fluid dynamics of tortuous blood vessels

Next, we investigated the inherent fluid dynamics in microchannels that met our design criteria with the aim to predict the propensity to enhance blood clotting due to vascular tortuosity. We imported the CAD drawings of tortuous microchannels to fluid modelling software (ANSYS), and performed computational fluid dynamic (CFD) simulations of blood flow assuming blood as a complex non-Newtonian fluid (see METHODS)^14^. Through this numerical modelling, we determined the hemodynamic profiles for varying tortuosity indices, that showed maximum velocity at the centre and no slip at the boundary as expected from the model (Figs. 1C, S1 and S2). However, these analyses revealed that the maximum wall shear stress increased with increasing tortuosity and imposed inlet velocity boundary condition (Fig. 1D). Importantly, our analysis of deviations from the mean across the channel showed that non-Newtonian flow in tortuous vessels also led to fluid shear gradients (acceleration and deceleration of flow) across the microchannel that also increased with tortuosity (Fig. 1E). These data thus predict that upon blood perfusion in a tortuous microchannel, the occlusion may be fastest if these channels were most tortuous. Based on this analysis, we decided to design a microfluidic device consisting channels of a tortuosity index, TI = 3.4. Further, since our analysis also showed that shear stresses and gradients increase upon increasing flow rates, we predicted more occlusion at high shear. However, in a single-pass device, a high flow rate may also lead to more blood consumption. Therefore, experimental optimization is required that leads to faster clotting due to high shear but keeps blood volume required low.

### Design of thrombus monitoring microfluidic device

**Figure 2:**
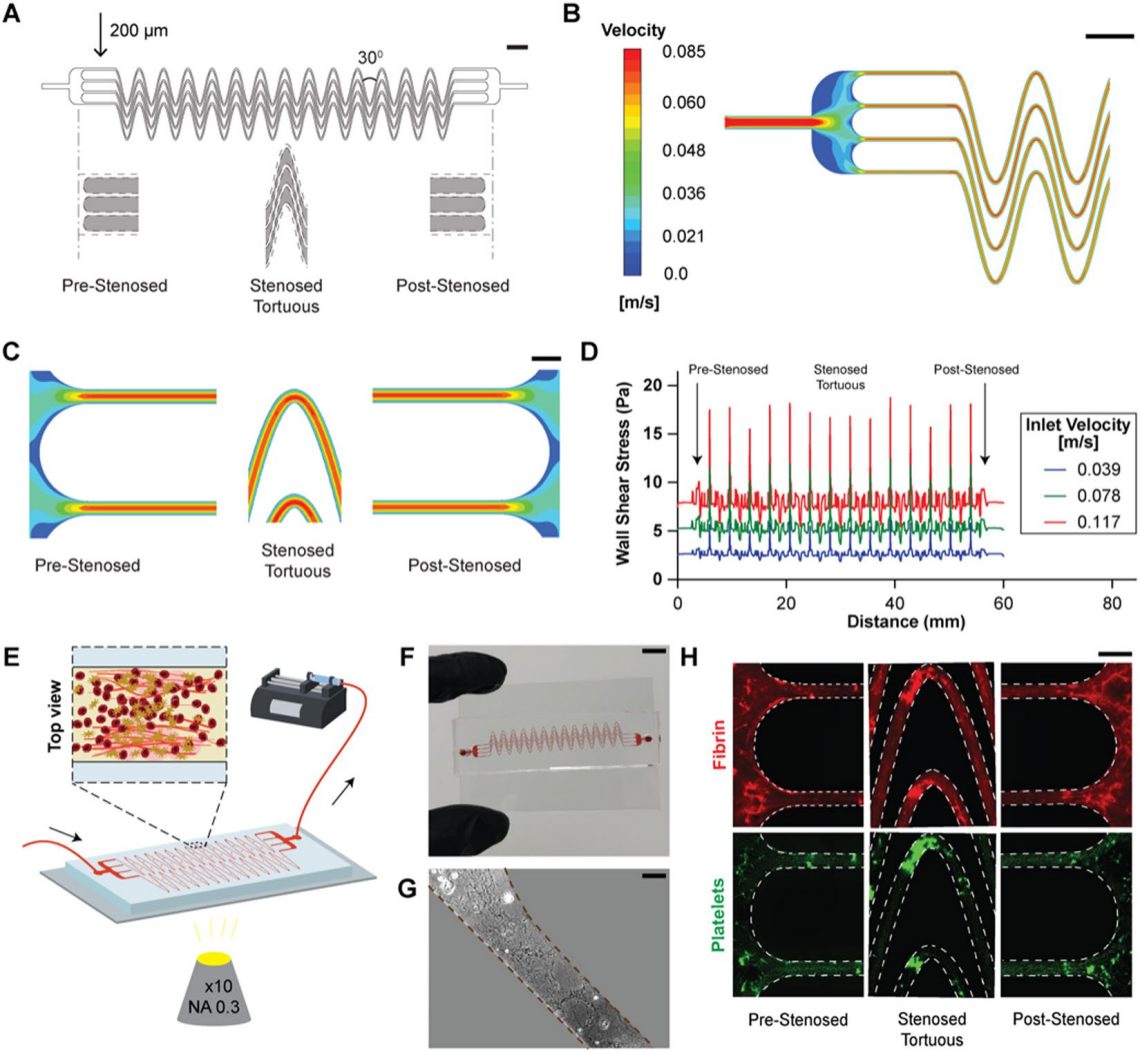
Design and characterization of tortuosity-powered microdevice. (**A**) Diagram of the device with line drawings (Solidworks v2019; www.solidworks.com) below showing the design of the accelerating (pre-stenosed), uniform region (stenosed tortuous) and decelerating (post-stenosed) regions of the microchannels. The central region contains 4 parallel stenosed tortuous lanes that are 200 µm wide by 75 µm high that repeatedly turn 30° frequently in each channel corresponding to TI = 3.4 (scale bar, 2 mm). (**B**) A representative heat map showing velocity (m/s) distribution in a large section of the device (scale bar, 2 mm), and (**C**) pre-stenosed (left), stenosed tortuous (centre), post-stenosed (right) sections of the device, at an inlet velocity of u = 0.078 m/s (scale bar, 400 µm). (**D**) Graph showing computed wall shear stress (Pa) across the length of the entire device (pre-stenosed through stenosed tortuous and post-stenosed; from left to right) at various inlet flow velocities. (**E**) Schematic of the microfluidic device mounted on a microscope and connected to a pump that pulls blood. An inline pressure sensor may be connected to the device via tubing and used to determine clotting time. (**F**) Photograph of the microfluidic device after an experiment (scale bar, 10 mm). The device is bonded on top of a collagen-coated standard glass slide (75 mm × 50 mm). **(G)** Representative image of rat tail collagen type I coating a section of the microdevice (scale bar, 100 µm). **(H)** Representative fluorescent micrographs of blood clots. Fibrin (top, red) and adhered platelets (bottom, green) in the pre-tortuous, stenosed tortuous, and post-tortuous regions of the device after perfusing re-calcified citrated whole blood over a rat collagen type 1 coated device (scale bar, 400 µm). CFD analysis was performed using Ansys (v19.2; www.ansys.com).

In prior work, we demonstrated a microfluidic device capable of inducing whole blood occlusion due to shear gradients caused by stenosis or converging-diverging channels^9^. Here, our objective was to design a microfluidic device that integrates the shear gradients induced by stenosis to tortuosity-driven gradients in the microdevice, so that blood clots could also form more rapidly and require lower blood volume. Therefore, we designed our device in a manner that upon blood perfusion, it mimicked stenosed tortuous arterioles to create sudden fluid acceleration (pre-stenosed), followed by a region of tortuosity and non-uniform shear (stenosed + tortuous), and then by a region with a sudden deceleration (post-stenosed; Fig. 2A, Movie S1). When we conducted a CFD analysis, it confirmed that the fluid undergoes several acceleration and deceleration stages along the device (Fig. 2B,C). Correspondingly, we found that the wall shear stress rapidly changes at the pre-tortuous and post-tortuous regions as shown in our previous study (Fig. 2D)^9^. But by introducing tortuosity, we saw that shear also fluctuates significantly in the tortuous region, creating a highly pathological and prothrombotic fluid mechanical environment within the device (Fig. 2D). Moreover, we saw that the absolute wall shear rate as well as its gradients increased with increasing inlet velocity, indicating that blood clot formation may be enhanced at higher flow rates. Thus, this design consisting three distinct shear gradient zones: pre-stenosed, stenosed + tortuous and post-stenosed together contribute to enhanced blood clotting. We then fabricated and mounted the device on the microscope for visualization and connected the device to the syringe pump to introduce flow (Fig. 2E). Blood first entered into a large reservoir (4.7 mm wide) and then flow split into four smaller parallel stenosed tortuous channels (200 µm wide); followed by convergence of the flow into an outlet similar to the inlet. The 4-channel design is partially analogous to a vessel network *in vivo* in which clots may form or detach locally, but the pressure still increases due to the systemic thrombosis. In addition, the total width and length of the device were designed to fit on a standard glass microscope slide for practical ease (Fig. 2F). Each device contains multiple single microchannel sections that were optimized to create maximum tortuosity (alternating 30° bends, corresponding to TI = 3.4), to expose flowing whole blood to varying shear rates due to both stenosis and tortuosity, thus promoting rapid clot formation and occlusion. To enable more rapid cell activation and adhesion, we further functionalized the surface of the microchannels within the device with collagen type I, which is a commonly applied platelet agonist^15^ (Fig. 2G). In practice, we operated the device at a flow rate of 70 µl min^−1^, leading to a pre-stenosed mean wall shear rate of 1, 200 s^−1^ which is typical arteriolar flow, and a wall shear gradient of 935 s^−1^mm^−1^ in the straight region following the pre-stenosed reservoir. Then, when we perfused re-calcified citrated whole blood through the microfluidic device for a maximum of 10 min at this flow rate, less than 1 mL of blood was consumed, and we detected formation of various sized thrombi throughout its entire length using fluorescence imaging (Fig. 2H). This suggested that this device could provide a tool to measure thrombosis in conditions requiring low blood volume and analysis within a few minutes. Importantly, these *in vitro* results indicate that physiologically-relevant whole blood thrombus formation may occur inside the device underlying a key advantage over standard laboratory tests that do not incorporate flow or require more specific coagulation pathways.

### Microdevice sensitivity to thrombin inhibitors evaluated via imaging

**Figure 3:**
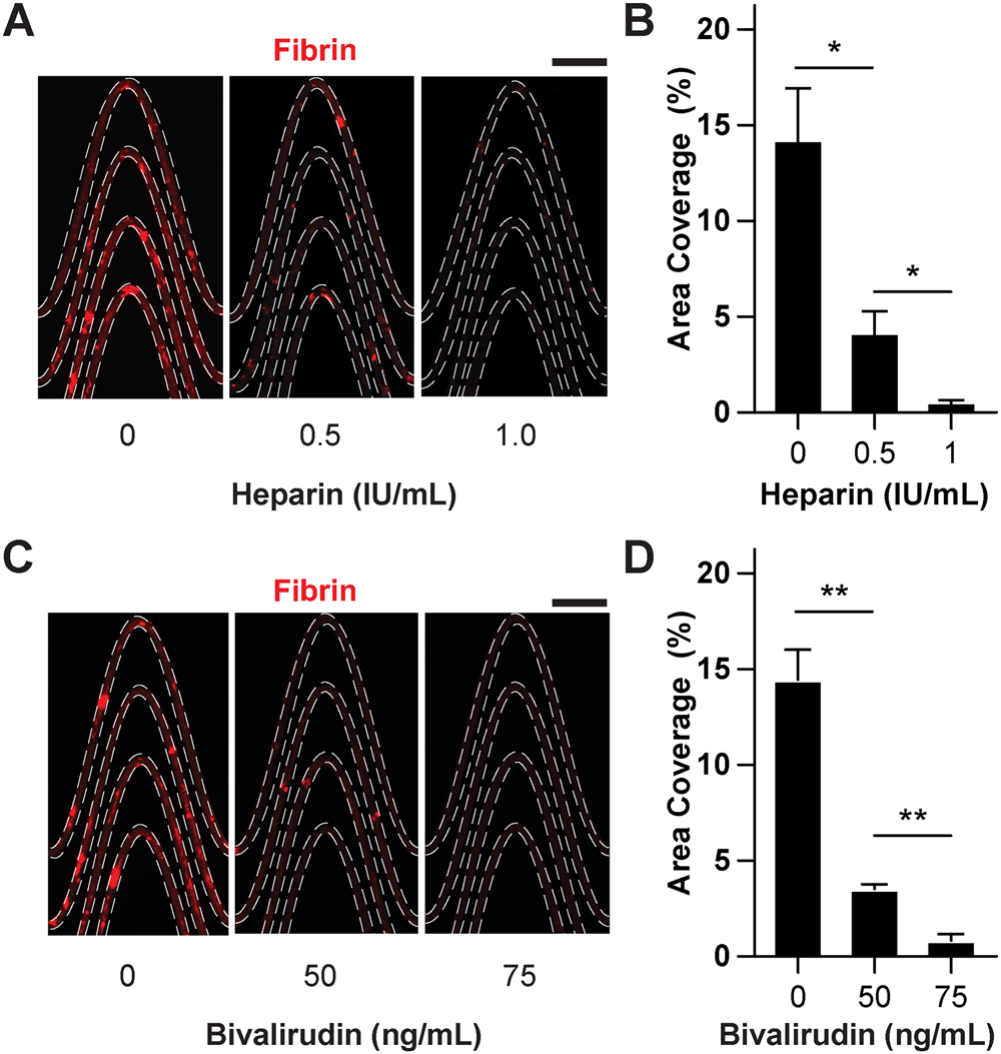
Analysis of thrombin inhibitors via imaging. **(A**) Representative fluorescent micrographs showing fibrin content within a section tortuous microchannels when blood consisted of heparin at various doses (scale bar, 1 mm). **(B)** Graph showing area coverage of fibrin at a section of the tortuous region for variable heparin doses. **(C)** Representative fluorescent micrographs showing fibrin content within a section tortuous microchannels when blood consisted of Bivalirudin at various doses (scale bar, 1 mm). **(D)** Graph showing area coverage of fibrin at a section of the tortuous region for variable bivalirudin doses. In (**A**,**C**), fibrin formed upon perfusion of re-calcified citrated whole blood treated with varying concentration of thrombin inhibitors (mean shear rate, 1,200 1/s) for 15 minutes (scale bar, 1 mm). In (**B**,**D**), *P < 0.05, **P < 0.01, unpaired t-test, SEM.; n = 3 donors.

Precise and personalized anticoagulant dose monitoring as close to a real-time basis is critical in patients on extracorporeal assist devices (for example, haemodialysis, membrane oxygenation, mechanical circulatory support, and so on) to ensure therapeutic anticoagulation, and to rapidly detect any life-threatening thrombotic or bleeding events that may arise^16^. To explore the potential utility of using this microfluidic device in monitoring the typical anticoagulants administered in critical care, we tested its sensitivity to unfractionated heparin (UFH), an indirect thrombin inhibitor. Also, UFH is the most commonly administered anticoagulant given to patients on extracorporeal assist devices^17^. First, when we added clinically-relevant doses of UFH (0–1 IU mL^−1^)^18^ to whole blood samples fluorescently labelled to track fibrin formation, and perfused the blood though the device while monitoring fibrin using fluorescence microscopy, we found decrease in fibrin area coverage as concentration of heparin was increased (Fig. 3A,B). These results suggested that this device can potentially detect differences in heparin dosage within blood samples *in-vitro*. Next, we explored if our device can also detect differences in doses of bivalirudin, a direct thrombin inhibitor, when added to blood samples. When we followed the same methodology as described above for heparin, but instead added clinically relevant doses of bivalirudin^19^ (0–100 ng mL^−1^) to whole blood, we again observed reduced fibrin with nearly complete clearance at 75 ng mL^−1^ (Fig. 3C,D) demonstrating a potentially unique advantage over current monitoring tools that have limited sensitivity to bivalirudin^20^.

### Microdevice sensitivity to thrombin inhibitors evaluated via pressure sensors

**Figure 4:**
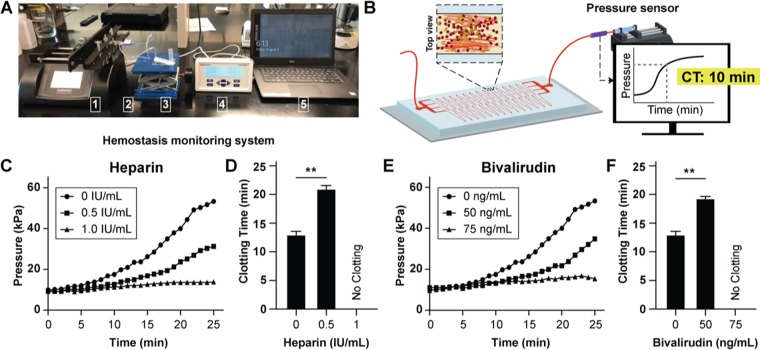
Analysis of thrombin inhibitors with in-line pressure sensor. (**A**) Photograph showing experimental setup that consists of a syringe pump (1) connected to an in-line pressure sensor (2), microfluidic device (3), pressure display monitor (4), and laptop computer (5). The syringe pump pulls the blood at a constant flow rate through the outlet of the microfluidic device and the changes in fluid pressure caused by channel occlusion are detected by the pressure sensor which sends the pressure data to the laptop computer for display. (**B**) Conceptual illustration of experimental set up and measurement of clotting time from pressure data. (**C**) Graph showing mean pressure trace measured in the device when re-calcified citrated whole blood treated with varying heparin doses (0, 0.5, 1.0 IU/mL) was perfused (mean shear rate, 1,200 1/s) through collagen coated devices (n = 3 donors, 1 replicate per experiment) (**D**) Clotting time (CT) derived from heparin pressure trace; (no clotting, **P < 0.01, n = 3 donors). (**E**) Graph showing mean pressure trace measured in the device when re-calcified citrated whole blood treated with varying bivalirudin doses (0, 50, 75 ng/mL) was perfused (mean shear rate, 1,200 1/s) (n = 3 donors, 1 replicate per experiment). (**F**) Clotting time (CT) derived from bivalirudin pressure trace; (no clotting, **P < 0.01, n = 3 donors).

Even though microscopic analysis of real-time thrombus formation in our blood thrombus monitoring device may be useful in labs, we were also inspired to potentially deploy this device at the point-of-care. Therefore, we connected this device to a syringe pump, disposable pressure sensor and a display monitor as we previously demonstrated^9^ (Fig. 4A). Since all of these components are already a part of most extracorporeal circuits, we expect this instrumentation to conveniently integrate in broad critical care settings^21^. Using this instrumentation, we can automatically track clot formation in real-time by measuring changes in fluid pressure caused by increasing channel occlusion inside the device (Fig. 4B). Importantly, we found that as recalcified citrated whole blood was perfused and formed clots inside the device, the pressure increases as was typical of the dynamics of clotting in blood vessel *in vivo*, or in an *in vitro* hollow channel, consisting of three stages - a steady reaction time, a growth phase, and saturation^22^. Correspondingly, when we added heparin (0–1 IU mL^−1^) into blood, we saw that the pressure rise shifted relative to normal controls as heparin concentration was increased (Fig. 4C,D). We saw a similar trend when we used bivalirudin (0–75 ng mL^−1^, Fig. 4E,F), demonstrating that this pressure sensor-based setup can detect effect of thrombin inhibitors *in-vitro* and potentially be used for monitoring them. Next, we developed a clotting time measurement for these pressure readouts to serve as a quantitative end point in lab and clinical settings. We set the 2.5X pressure rise from baseline measurement as an endpoint and found that this metric of clotting time decreased as concentration increased for both heparin and bivalirudin (Fig. 4D,F). Importantly, we found that channel occlusion occurred within 13 minutes using less than 1 mL of whole blood in untreated samples, an approximately 35% improvement over the previous microfluidic assay^9^, which indicates that the introduction of tortuosity-driven shear gradients accelerated clot formation.

### Microdevice sensitivity to platelet count

**Figure 5:**
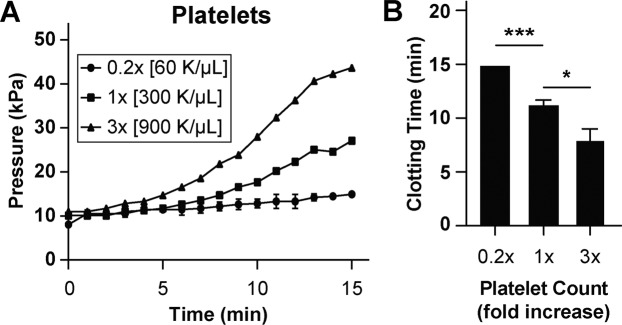
Platelet count analysis. (**A**) Graph showing mean pressure trace measured through the device when re-calcified citrated whole blood containing varying platelet counts was perfused (mean shear rate, 1,200 1/s) through devices for 15 minutes (n = 3 donors). **(B)** Clotting time (CT) derived from pressure trace measurements (***P < 0.001, *P < 0.05, n = 3 donors).

As platelet count can be a major contributor to the development of vascular occlusion in many clinically settings, we further explored if this biomimetic device can be used to detect changes in clotting time due to platelet count under stenosed and tortuous flow^23^. First, we measured platelet adhesivity in devices coated with collagen (type I, rat, 100 µg mL^−1^) using recalcified whole blood with varying platelet counts to explore if this device can potentially be used to predict disorders where platelet count is low or elevated. We produced blood samples with 0.2X and 3X platelet counts relative to normal controls (1X) and when these samples were perfused, we observed a relative shift of pressure curves to the right and left for the 0.2X and 3X samples, respectively (Fig. 5A). Also, when these pressure traces were measured for clotting time, we observed a dose-dependent decrease in clotting time as a function of increasing platelet count (Fig. 5B). These results validate that platelets are a critical component of occlusion within this device since their removal and addition to blood affected clotting dynamics and time. This also provides a proof-of-concept that this device could potentially be used for predicting conditions associated with platelet count or therapy such as, platelet transfusion.

### Analysis of anti-fibrinolytics

**Figure 6:**
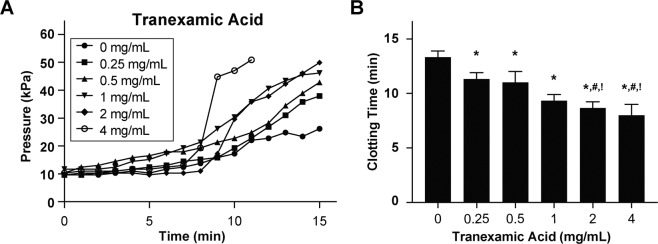
Analysis of antifibrinolytics. (**A**) Graph showing mean pressure trace measured through the device when re-calcified citrated whole blood containing varying doses of Tranexamic Acid was perfused (mean shear rate, 1,200 1/s) through devices for 15 minutes (n = 3 donors). (**B**) Clotting time (CT) derived from pressure trace measurements (*P < 0.05 against 0 mg/mL, ^#^P < 0.05 against 0.25 mg/mL, ^!^P < 0.05 against 0.5 mg/mL; n = 3 donors).

Patients on extracorporeal mechanical systems can experience hyperfibrinolysis and as a result, are sometimes administered antifibrinolytics to decrease the risk of having a bleeding episode^24,25^. However, the use of these drugs are debatable partly because there are no assays that can provide reliable diagnostics of impact of delivering these drugs to patients^26^. To explore the potential utility of this device for monitoring antifibrinolytic therapy, we tested its sensitivity to clinically-relevant doses of tranexamic acid (Cyklokapron™, 0–4 mg mL^−1^), a synthetic lysine amino acid derivative which acts as an inhibitor of plasminogen activation or plasmin^27^. We perfused whole blood containing increasing doses of tranexamic acid and observed a relative shift in pressure curve to the left as we increased the dosage of drug samples and corresponding decrease in clotting time (Fig. 6A,B). These results suggest that the addition of this drug is preventing fibrin degradation due to plasminogen or plasmin present in blood and promoting clotting inside the microchannels correspondingly. Furthermore, this also demonstrates that this device could potentially be used to monitor antifibrinolytic therapy in practical settings where hyperfibrinolysis arises.

### Assessment of human paediatric patients in critical care

**Figure 7:**
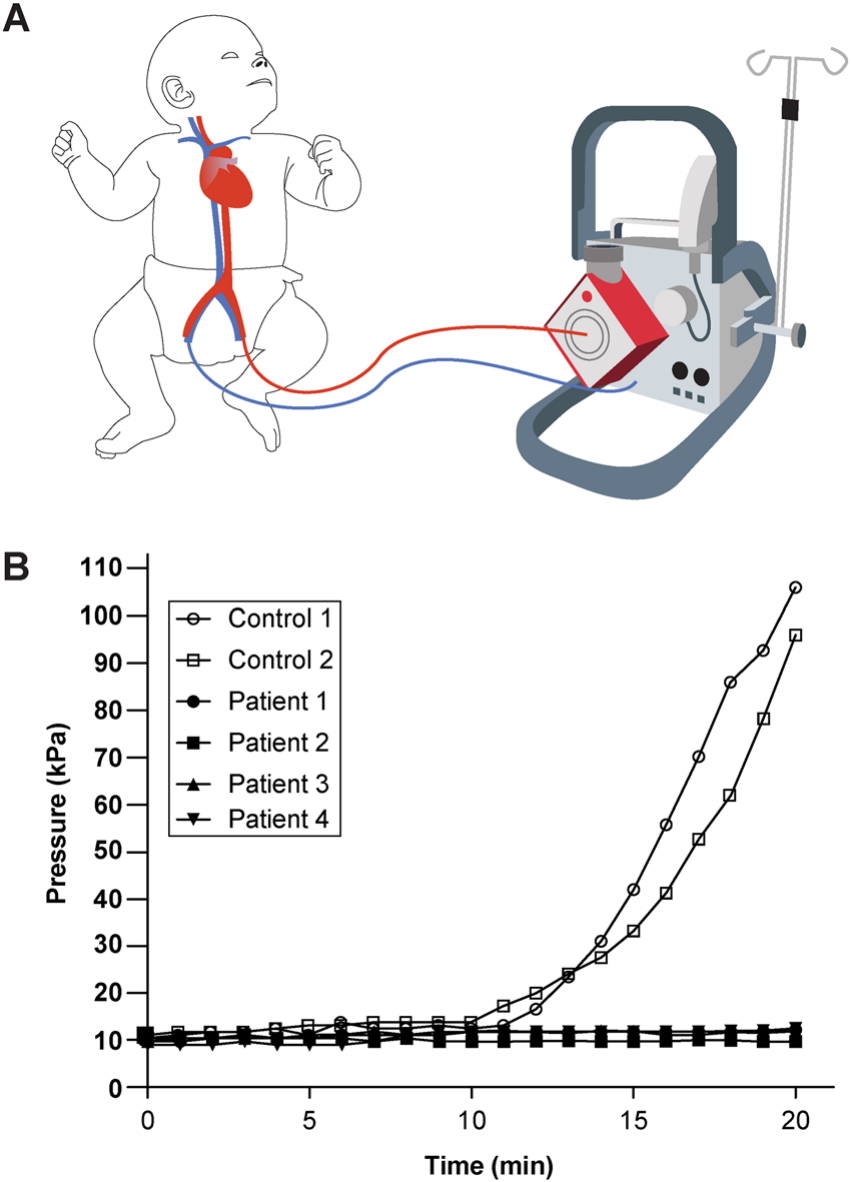
Analysis of blood samples of paediatric patients on ECMO. (**A**) Clotting time analysis was performed with blood samples obtained from paediatric patients on ECMO. (**B**) Graph showing pressure trace measured in the device when re-calcified citrated whole blood from paediatric healthy and ECMO patients was perfused through devices for 20 minutes (n = 2 normal and 4 ECMO donors).

Paediatric patients on extracorporeal life support systems typically have low platelet counts, platelet dysfunction, acquired von Willebrand syndrome, hyperfibrinolysis and loss of coagulation factors^28,29^. As a result, they are highly susceptible to dramatic alteration in normal haemostasis (Fig. 7A). We explored whether we could use this device to measure defects in haemostasis present in blood samples from paediatric patients on ECMO receiving anticoagulation. The patients were receiving heparin therapy, and were diagnosed with bleeding symptoms clinically, as well as low platelet counts (Table S1). Interestingly, when we perfused blood from four pediatric ECMO patients within the device that was designed to cause rapid clots, we did not observe any clotting for a duration of 20 minutes, clearly suggesting that due to the combined effect of disease, surgery, and therapy, their haemostasis was significantly abnormal (Fig. 7B). In contrast, healthy subjects exhibited increase in pressure and thrombosis within the device. These data suggest that this microfluidic device can potentially be applied to test patients in critical care with altered haemostasis due to combined effects of anticoagulation and other likely acquired symptoms due to operating procedures.

## Discussion

This study was designed with the hypothesis that physiological relevance of a platform that measures blood clotting will potentially serve as a more reliable point-of-care device. Several past studies, including our own prior work, have illustrated that coagulation tests, such as the ACT and aPTT, which do not include a physiologically-relevant microenvironment (for example, fluid shear or fluid shear gradients), have limited predictive power^9,30^. Moreover, although viscoelastic methods, such as TEG and ROTEM, do assess whole blood clotting, the thrombus measured are still mostly activated using artificial reagents, such as kaolin and phospholid^31^. Here, we extended the prior art by introducing another novel and underexplored hemodynamic parameter, tortuosity. The tortuosity-activated microfluidic device described here provides several potential advantages over other tests used to monitor haemostasis and thrombosis. First, due to the integration of complex vascular architecture (stenosis and tortuosity) in the microfluidic design, clotting inside the device is activated by a pathophysiologically-relevant fluid mechanical environment and includes complex blood rheology that is critical to thrombosis. Another major advantage of this tortuosity-activated device is that when connected to a pressure sensor, blood clots can be quantified. The clotting time derived from the pressure curve can detect differences in blood spiked with clinically-relevant doses of anticoagulants (thrombin inhibitors) and antifibrinolytics (tranexamic acid) *in-vitro*, and it shows sensitivity to platelet count under stenosed and tortuous flow, making this device potentially well suited to explore whether anticoagulants, antifibrinolytics and anti-platelet drug candidates that produce different behaviours in clotting dynamics. Importantly, we were able to confirm that paediatric patients on ECMO as well as anticoagulants did not clot within our device. Therefore, this data provides a proof-of-feasibility that device may be used to identify changes in haemostasis at bedside and guide therapy.

This device also has limitations that may be addressed in future studies. For instance, our device does not entirely mimic physiological blood vessel architecture and blood flow, since *in vivo* blood flow is pulsatile, and the walls are cylindrical and elastic. Second, the time to assess clotting is still in the order of several minutes. But most existing haemostasis assays are also not instantaneous, and clinical decisions are often made over the time span of many hours. Thus, the 10–15 minutes that are required for completion of this assay by the bedside using disposable analytical components should still be advantageous compared to the multiple blood draws and dependence on lab facilities in the hospital. However, for this to be achieved will require extensive collaboration with hospitals that can provide considerable resources (coordinating staff, patients, funding) to demonstrate feasibility for these various indications.

In addition, our purpose was not to reveal the mechanisms that govern thrombosis *in vivo* per se, rather engineer a microfluidic device that utilizes tortuosity-driven thrombotic processes to form clots more rapidly and requiring lower blood volume in such measurements. However, since the device does offer tunability in design, it could also be used to more accurately mirror blood vessels and provide in-depth understanding of mechanisms.

In conclusion, these preliminary findings suggest that this tortuosity-activated device may be useful in serving as a tool to diagnose clotting disorders and guide therapy where thrombotic or antithrombotic drugs are administered. In future, larger datasets constituting samples taken from patients in clinic will be needed to establish the specificity and sensitivity of this assay against various scenarios of clotting.

## Methods

### Device fabrication and thrombus monitoring

The method pertaining to device design and integration with instrumentation to measure pressure is presented in detail in our prior work^9^. Briefly, we fabricated the devices from PDMS soft lithography to a size that would fit on a standard (75 × 50 mm) microscope slide^32^. The cured PDMS from the master mold was bonded to a 500-mm-thick PDMS-coated glass slide and punched with inlet and outlet 1.5 mm holes. Next, we coated the device with rat tail type I collagen (Corning) at 100 µg mL^−1^ by infusing into the device pre-treated with silane. The devices were incubated at 37 °C for 3–4 h and washed with saline solution before blood perfusion. At the inlet side, a reservoir was created by force-fitting an open slip-tip syringe and fresh blood was stored. At the outlet, tubing was connected to an inline, disposable pressure sensor (PendoTECH) which provided the information of pressure change across the device. The other end of the sensor was connected to a syringe pump that pulled the blood at a mean arteriole wall shear rate of 1200 s^−1^. Thrombus formation was observed using time-lapse imaging (10X, NA 0.35) of fluorescently labelled fibrinogen (15 µg mL^−1^, Alexa Fluor 647, Invitrogen) and platelets (10 µL mL^−1^ Human CD41-PE (clone: VIPL3), Invitrogen) added directly to the blood and incubated at room temperature for 8 min for whole blood imaging. Fluorescence microscopy of fibrin and platelets was then performed at an interval of every 30 seconds.

### Computational fluid analysis

To simulate blood flow and analyse wall stresses and shear rates, we used a finite element-based software, Ansys Fluent (v19.2, www.ansys.com). The drawing of the entire device made in Solidworks (v2019) software was exported to Ansys, and Navier-Stokes equations were solved. Blood was assumed to be a two-dimensional, incompressible, non-Newtonian fluid. A finite velocity as the inlet boundary condition and constant atmospheric pressure (P = 0) at the outlet boundary were assigned to the model. To describe the non-Newtonian behaviour of blood, a generalized power-law constitutive equation for viscosity was applied with parameters based on published values^14^.

### Blood samples

Blood from healthy adult donors was collected upon informed consent in 3.2% sodium citrate tubes (BD Biosciences). After receiving informed consent from parents or legal guardians, specimens were also collected from paediatric patients on extracorporeal membrane oxygenation (ECMO) via the circuit in 3.2% sodium citrate tubes (Greiner Bio-One). The studies were approved and conducted in accordance with the Texas A&M University Institutional Review Board (IRB ID: IRB2016-0762D) and Baylor College of Medicine Institutional Review Board (IRB ID: H-27793). Blood was used within 2–4 hours of withdrawal to prevent abnormal platelet functioning^33^. The coagulation activity of these samples was restored by adding 100 μL mL^−1^ of 100 mM calcium chloride/75 mM magnesium chloride solution.

### Platelet count

Platelet count was measured using a complete blood count (CBC) machine (Erba Diagnostics, Hemavet). Platelets were first separated from blood via standard centrifugation methods^34^ and they were added back to blood to achieve required concentration (count/mL).

### Drugs

Drugs were purchased from Texas A&M pharmacy. Heparin, bivalirudin, or tranexamic acid (Cyklokapron) were dissolved in the blood to required concentrations respectively.

### Statistical analysis

*In vitro* assay sample size was predetermined with three separate donors to account for biological variability. Data analysis were performed using GraphPad Prism V7. Statistical t-tests were conducted to compare experimental groups. The data are presented as mean and standard error of mean (SEM).

## Supplementary information

Supplementary Information.

Supplementary Movie.
