# Supplementary material for: Tortuosity-powered microfluidic device for assessment of thrombosis and antithrombotic therapy in whole blood
Source: Sci Rep. 2020 Apr 1;10:5742. doi: 10.1038/s41598-020-62768-4 (PMC7113244; doi:10.1038/s41598-020-62768-4)
Supplement: Supplementary file 1 — Supplementary Information. [file 41598_2020_62768_MOESM1_ESM.docx]

**Tortuosity-powered microfluidic device for assessment of thrombosis and haemostasis in whole blood**

David J. Luna ^a^, Navaneeth K.R. Pandian ^a^, Tanmay Mathur ^a^, Justin Bui ^a^, Pranav Gadangi ^a^, Vadim V. Kostousov ^b^, Shiu-Ki Hui ^b,c^, Jun Teruya ^b,c,d^ and Abhishek Jain ^a,e^*

^a^Department of Biomedical Engineering, Texas A&M College of Engineering, College Station, TX.

^b^Division of Transfusion Medicine & Coagulation, Department of Pathology & Immunology, Texas Children’s Hospital & Baylor College of Medicine, Houston, TX.

^c^Department of Paediatrics, Texas Children’s Hospital & Baylor College of Medicine, Houston, TX.

^d^Department of Medicine, Texas Children’s Hospital & Baylor College of Medicine, Houston, TX.

^e^Department of Medical Physiology, College of Medicine, Texas A&M Health Science Center, Bryan, TX

**SUPPLEMENTARY INFORMATION**

**Supplementary Figures**

**
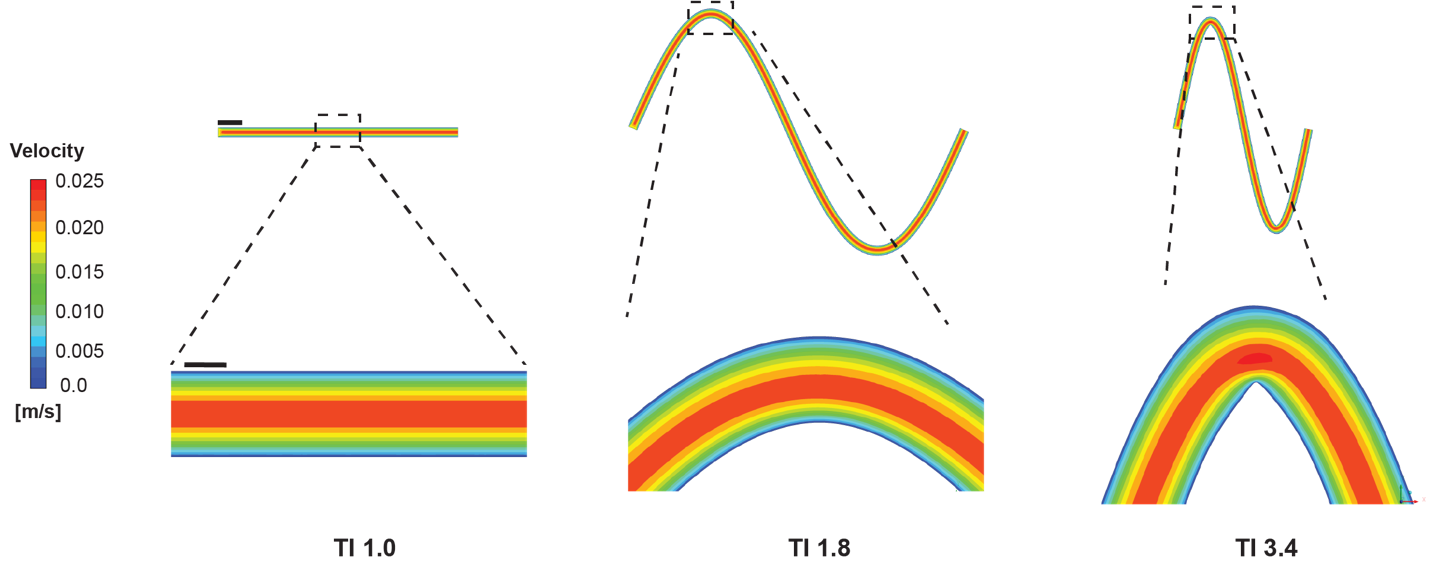
**

**Supplementary Fig 1.** Velocity profiles in tortuous microchannels. Representative heat maps derived from CFD analysis showing the velocity profile in TI 1.0, 1.8, and 3.4 microchannels (scale bar, 500µm) and curve (scale bar, 100µm) at an inlet velocity of u = 0.017 m/s (scale bar, 100µm).

**
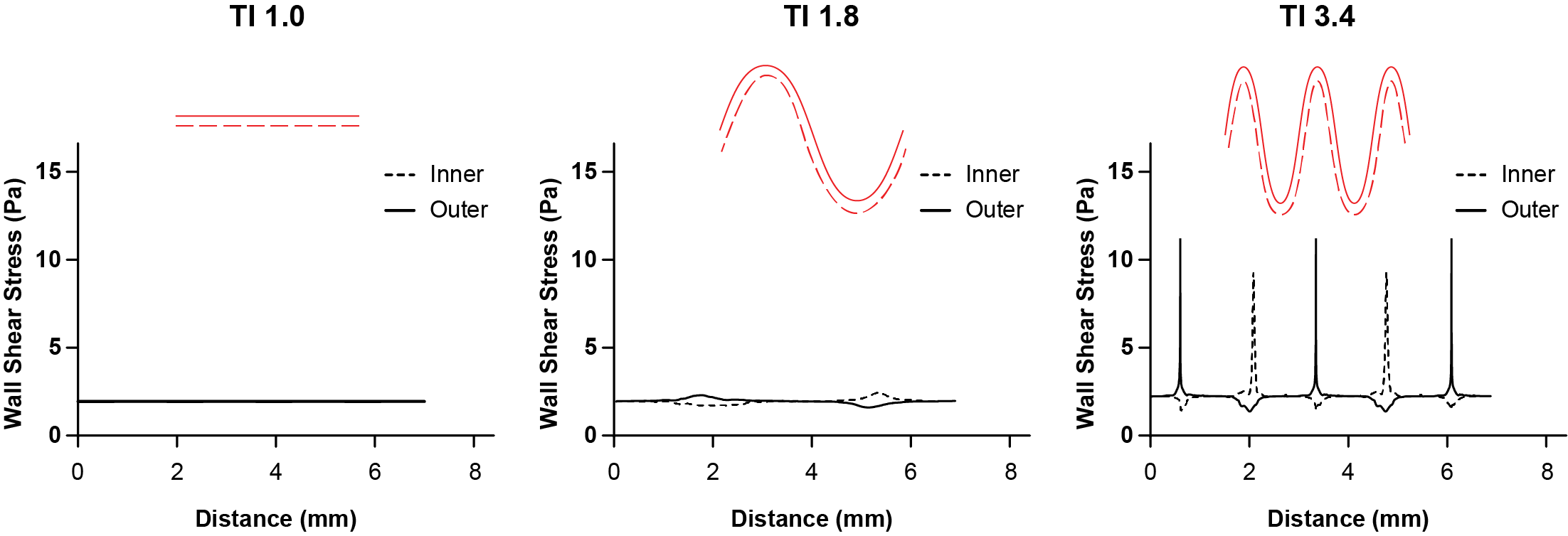
**

**Supplementary Fig 2.** Wall shear stress distribution in tortuous microchannels. Representative plots showing wall shear stress profile for TI 1.0, 1.8, and 3.4 microchannels at an inlet velocity of u = 0.017 m/s.

**Supplementary Movies**

**Movie 1**. **Whole blood flow in microdevice.** Brightfield video of whole blood flow in the microchannels of the tortuosity activated device.

**Supplementary Table**

| **Pediatric Patient** | **Hb**  **(11.5 – 15.5 g/dL)** | **HCT (35 – 45%)** | **Platelets (150 – 450)** |
| --- | --- | --- | --- |
| Control 1 | 14.1 | 42.2 | 389 |
| Control 2 | 12.1 | 39.7 | 314 |
| Patient 1 | 9.8 | 29.0 | 125 |
| Patient 2 | 11.4 | 35.0 | 131 |
| Patient 3 | 12.4 | 38.1 | 153 |
| Patient 4 | 11.5 | 35.2 | 176 |

**Supplementary Table 1. Complete blood count parameters for pediatric controls and ECMO patients.** Table showing complete blood counts results for 2 pediatric control and 4 pediatric ECMO patients.
